# TRAF2 in osteotropic breast cancer cells enhances skeletal tumour growth and promotes osteolysis

**DOI:** 10.1038/s41598-017-18327-5

**Published:** 2018-01-08

**Authors:** Prabha Peramuhendige, Silvia Marino, Ryan T. Bishop, Daniëlle de Ridder, Asim Khogeer, Isabella Baldini, Mattia Capulli, Nadia Rucci, Aymen I. Idris

**Affiliations:** 1 Department of Oncology and Metabolism, University of Sheffield, Medical School, Beech Hill Road, Sheffield, S10 2RX UK; 2 Bone and Cancer Group, Edinburgh Cancer Research Centre, MRC Institute of Genetics and Molecular Medicine, University of Edinburgh, Edinburgh, EH4 2XR UK; 3 University of L’Aquila, Department of Biotechnological and Applied Clinical Sciences, L’Aquila, Italy

## Abstract

NFκB plays an important role in inflammation and bone remodelling. Tumour necrosis factor receptor associated factor 2 (TRAF2), a key component of NFκB signalling, has been identified as an oncogene, but its role in the regulation of breast cancer osteolytic metastasis remains unknown. Here, we report that stable overexpression of TRAF2 in parental and osteotropic sub-clones of human MDA-MB-231 (MDA-231) breast cancer cells increased cell growth and motility *in vitro*, whereas TRAF2 knockdown was inhibitory. *In vivo*, TRAF2 overexpression in the parental MDA-231-P cells enhanced tumour growth after orthotopic injection into the mammary fat pad of mice but failed to promote the metastasis of these cells to bone. In contrast, overexpression of TRAF2 in osteotropic MDA-231-BT cells increased skeletal tumour growth, enhanced osteoclast formation and worsened osteolytic bone loss after intra-tibial injection in mice. Mechanistic and functional studies in osteotropic MDA-231-BT and osteoclasts revealed that upregulation of TRAF2 increased the ability of osteotropic MDA-231-BT cells to migrate and to enhance osteoclastogenesis by a mechanism dependent, at least in part, on NFκB activation. Thus, the TRAF2/NFκB axis is implicated in the regulation of skeletal tumour burden and osteolysis associated with advanced breast cancer.

## Introduction

TNF receptor associated factor (TRAF) super family of adaptor proteins plays an important role in inflammation, breast cancer and bone remodelling^1–4^. Of the 7 known members of the TRAF superfamily, TRAF2 is a common target for pro-inflammatory and tumour-derived factors such as tumour necrosis factor alpha (TNFα), interleukin 1 (IL-1), receptor activator of NFκB ligand (RANKL), transforming growth factor beta (TGFβ), and cluster of differentiation 40 ligand (CD40L)^2,3,5–9^. Binding of these mediators to their receptors initiates the recruitment of TRAF2 to their respective receptors^2,6,7,10–14^ and triggers the activation of multiple intracellular signalling cascades including the canonical NFκB pathway^2,5–8,15,16^. TRAF2 also undergoes phosphorylation, protein–protein interaction with DNA and other TRAF molecules and functions as an E3 ubiquitin ligase to activate downstream events essential for NFκB activation^3,15–19^.

The TRAF2/NFκB signalling pathway plays a critical role in lactating mammary-gland development and breast cancer. Elevated TRAF2 expression correlates with breast cancer cell invasion and metastasis in patients^8,20^. In addition, TRAF2 regulates the activity of various breast cancer oncogenes, and inhibition of TRAF2 has been associated with reduction in mammary tumour growth^6,10,12,17,19,21–23^. Recently, TRAF2 has been described as an NFκB activating oncogene in breast epithelial cells^6,7^. Breast cancer cells in bone (osteotropic) cause bone damage by producing osteolytic factors that enhance osteoclastogenesis and inhibit osteoblast differentiation via NFκB activation^24–26^. TRAF2-mediated NFκB activation plays a key role in the regulation of bone remodelling, and numerous studies have demonstrated that inflammation-induced TRAF2 activation causes bone loss by increasing osteoclast formation and inhibiting osteoblast survival^2,4,26–28^. However, the role of TRAF2 in the development of breast cancer bone metastasis and its contribution to osteoclast and osteoblast changes associated with these metastases has not been investigated.

In the present study, we showed that cancer-specific TRAF2 expression contributes to breast cancer-induced osteolysis. Our *in vivo*, *ex vivo* and *in vitro* investigation showed that TRAF2 expression in the osteotropic MDA-231 human breast cancer cells increases tumour cell growth in bone, and enhances the ability of these cells to induce osteoclast formation and to cause osteolysis in mice. Thus, therapeutic targeting of TRAF2/NFκB signalling may be of value in protecting the skeleton from osteolytic bone damage associated with advanced breast cancer.

## Results

### Upregulation of TRAF2 enhances parental tumour cell growth *in vitro* and *in vivo*

**Figure 1:**
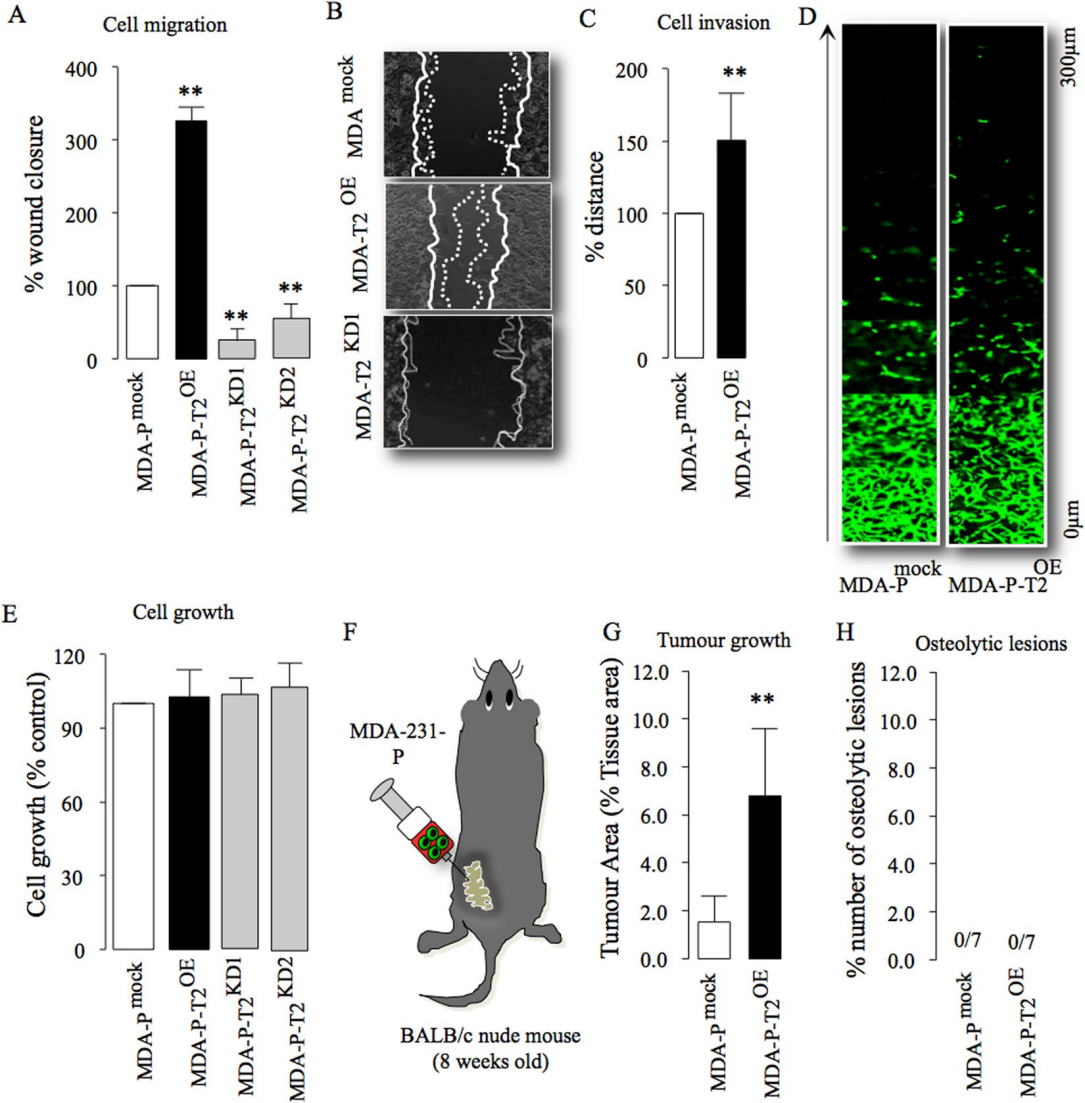
TRAF2 enhances breast cancer tumour cell growth *in vitro* and *in vivo*. (**A,B**) *In vitro* cell migration of parental human MDA-231 (MDA-P) cells overexpressing (MDA-P-T2^OE^) and deficient (MDA-P-T2^KD1^ and T2^KD2^) in TRAF2 or their control (MDA-mock) after 6 hours as assessed by wound healing assay. Representative photomicrographs from the experiment described are shown in panel B. (**C**,**D**) *In vitro* cell invasion of parental MDA overexpressing TRAF2 (MDA-P-T2^OE^) cells or their control (MDA-P^mock^). Representative photomicrographs from the experiment described are shown in panel D. (**E**) *In vitro* cell viability of parental human MDA-P cells overexpressing (MDA-P-T2^OE^) and deficient (MDA-P-T2^KD1^ and T2^KD2^) in TRAF2 or their control (MDA-mock) after 48 hours as assessed by AlamarBlue assays. (**F**) Graphic representation of orthotropic injection of parental human MDA-231 overexpressing TRAF2 cells into the mammary fat pads of adult mice (n = 7, 55 days). (**G**) *In vivo* tumour growth from the experiment described in panel F. (**H**) Percentage bone metastases from the experiment described in panel F. **p < 0.01.

TRAF2 enhances mammary tumour growth^7^, and we have observed that TRAF2 expression is upregulated in the human osteotropic MDA-231 breast cancer cell line when compared to their parental control (Fig. S1A). In view of this, we hypothesized that TRAF2 is implicated in bone metastasis, skeletal tumour growth and osteolysis associated with advanced breast cancer. We stably overexpressed (Fig. S1B,C) and knocked down (Fig. S1D,E) TRAF2 in the parental MDA-231-P and their osteotropic sub-clone MDA-231-BT (Fig. S1B–E), and assessed the growth and metastatic behaviour of these cells *in vitro*, *ex vivo* and *in vivo*. First, we confirmed that TRAF2 overexpression in parental MDA-231-P enhanced migration within 6 hours, whereas its knockdown in these cells was inhibitory (Fig. 1A,B). Overexpression of TRAF2 also increased the invasion of parental MDA-231-P after 48 hours (Fig. 1C,D). Of note, TRAF2 overexpression or knockdown had no effect on the viability of parental MDA-231-P (Fig. 1E). Next, we evaluated the mammary tumour growth and bone metastasis of parental MDA-231-P in mice after orthotopic injection (Fig. 1F,H). Overexpression of TRAF2 enhanced MDA-231-P growth in the mammary fat pads (Fig. 1G) but failed to cause osteolytic lesions in mice (Fig. 1H). We also failed to detect any MDA-231-P cells expressing green fluorescent protein in bone aspirates *ex vivo* (data not shown).

### Upregulation of TRAF2 enhances breast cancer-induced osteolysis *in vivo*

**Figure 2:**
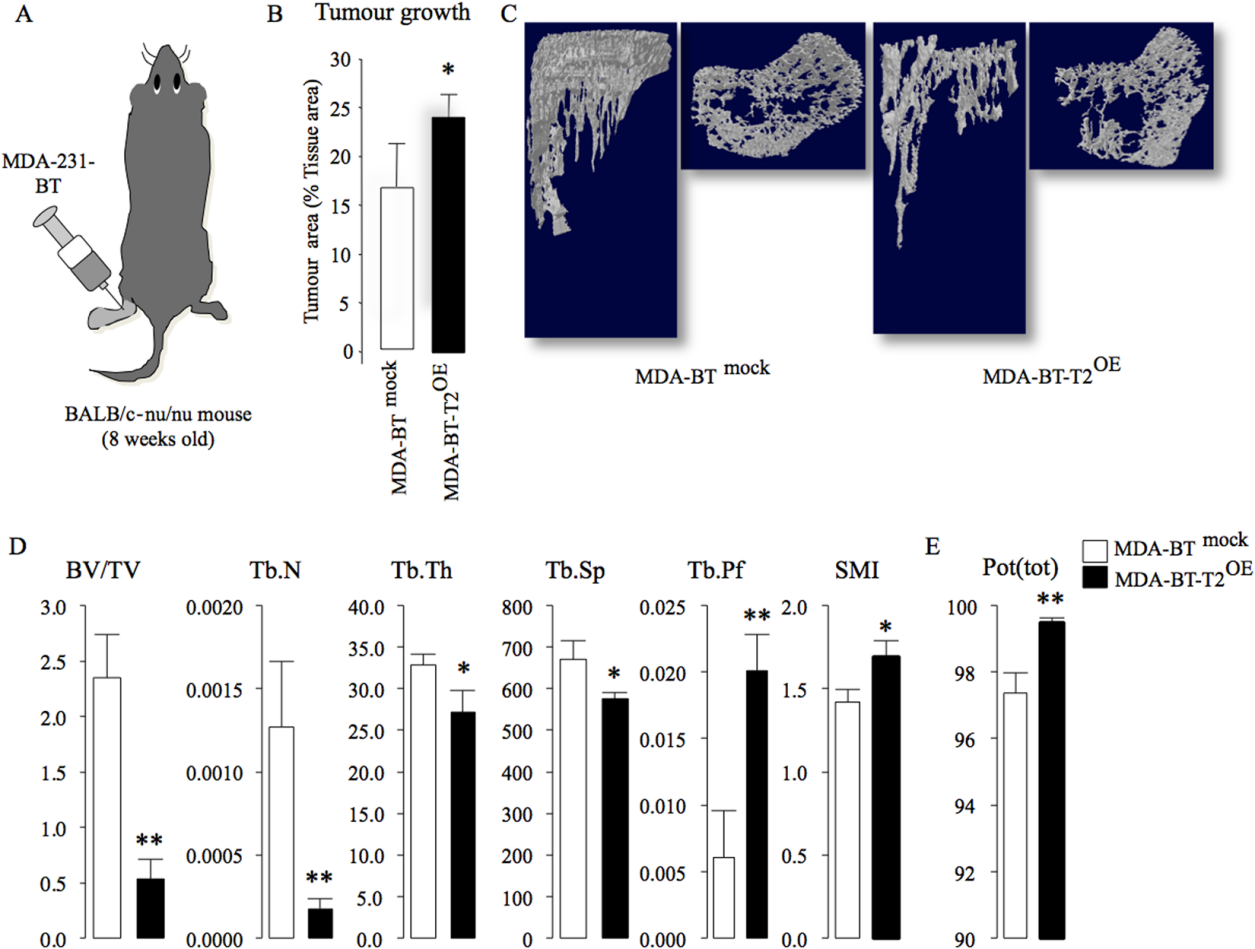
TRAF2 enhances skeletal tumour growth and osteolysis in mice. (**A**) Graphic representation of intra-tibial injection of the osteotropic human MDA-231-BT overexpressing TRAF2 (MDA-BT-T2^OE^) cells or their control (MDA-BT ^mock^) (n = 7, 14 days) in adult BALB/c‐nu/nu athymic mice. (**B**) *In vivo* tumour area in bone (% tissue area) from the experiment described in panel A. (**C**) Representative microCT scans of tibial metaphysis of mice from the experiment described in panel A. (**D**) *In vivo* trabecular bone volume (BV/TV, %), Trabecular number (Tb. N, 1/μm), Trabecular thickness (Tb.Th, μm), Trabecular separation (Tb.Sp, μm), Trabecular pattern factor (Tb.Pf, 1/μm) and structure model index (SMI) in tibial metaphysis from the experiment described in panel A. (**E**) *In vivo* cortical porosity (Pot(tot), %) from the experiment described in panel A. *p < 0.05, **p < 0.01.

To test if TRAF2 is implicated in the regulation of osteolytic activity of breast cancer cells, we assessed skeletal tumour growth and osteolysis in mice after intra-tibial injection of the osteotropic sub-clone MDA-231-BT. Overexpression of TRAF2 in these cells markedly increased tumour growth in bone (Fig. 2A,B) and enhanced their ability to cause osteolysis (Fig. 2C,E). As shown in Fig. 2, panel D, detailed microCT analysis of bone samples from this experiment revealed significant reduction in trabecular bone volume (BV/TV) that is accompanied by reduced trabecular number and thickness, increased trabecular separation and decreased trabecular connectivity (increased trabecular pattern factor and SMI). We also detected significant increase in cortical porosity (Fig. 2E) whereas cortical area and thickness remained unchanged (data not shown).

### Cancer-specific TRAF2 regulates osteotropic breast cancer – bone cell crosstalk

**Figure 3:**
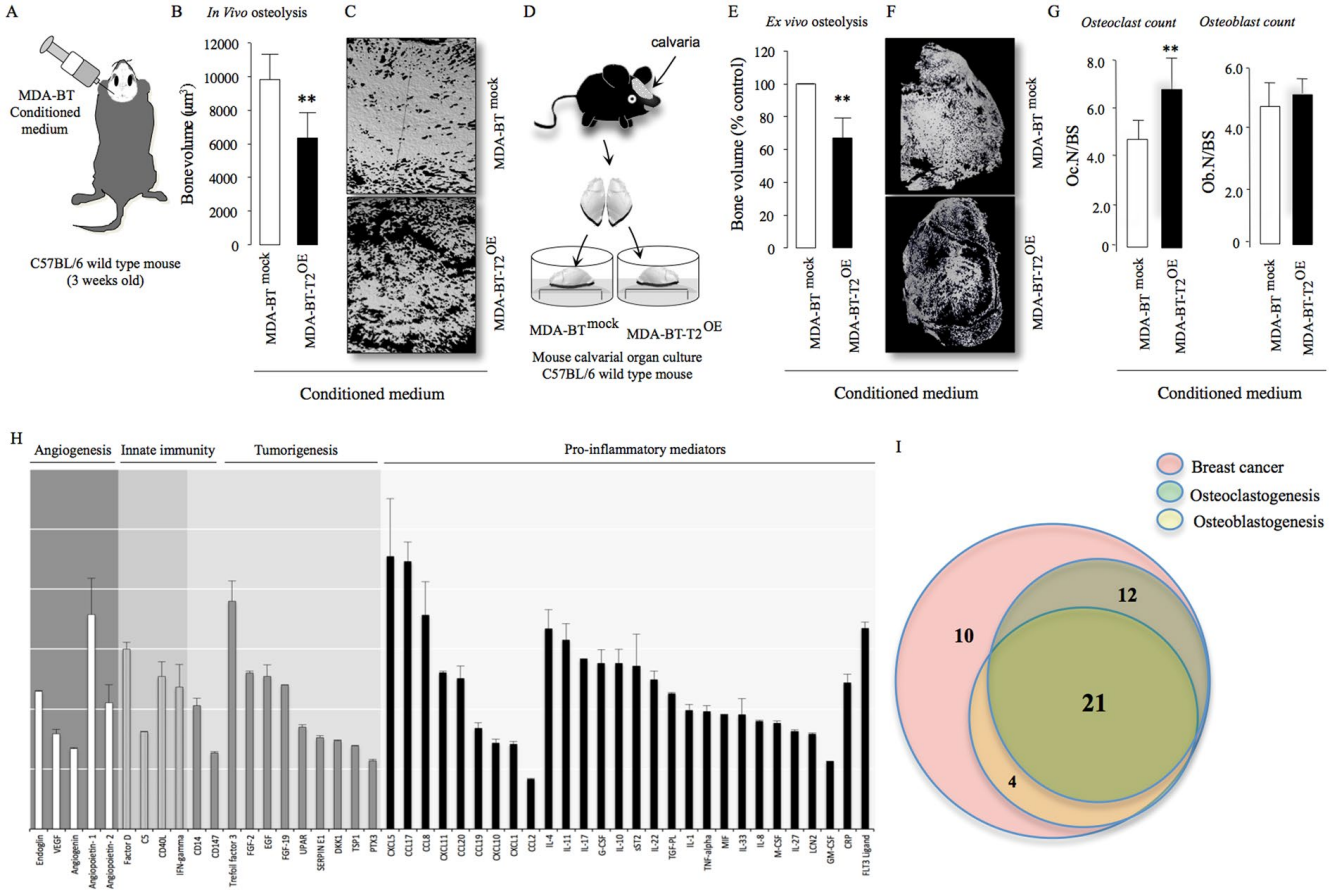
TRAF2 enhances level of tumour-derived osteolytic factors. (**A**) Graphic representation of supracalvarial injection of conditioned medium (CM) from the osteotropic human MDA-231 overexpressing TRAF2 (MDA-BT-T2^OE^) cells or their control (MDA-BT ^mock^) (n = 7, 5 days) in immunocompetent mice. (**B**) *In vivo* osteolysis from the experiment described in panel A. (**C**) Representative microCT scans of mouse calvarial bone from experiment described in A. (**D**) Graphic representation of mouse calvarial organ co-culture system. (**E**) *Ex vivo* osteolysis as measured by loss of bone volume in mouse calvaria bone after exposure to conditioned medium (20% v/v) from the osteotropic human MDA-231 overexpressing TRAF2 (MDA-BT-T2^OE^) cells or their control (MDA-BT ^mock^) (n = 8, 7 days). (**F**) Representative microCT scans of mouse calvaria bone from experiment described in D. (**G**) Osteoclast and osteoblast numbers from the experiment described in D. (**H**) Catalogue of differentially expressed inflammatory mediators in conditioned medium from mock control or TRAF2 overexpressing (MDA-BT-T2^OE^) human breast cancer cells as assessed by Proteome Profiler Human XL Cytokine Array Kit. (**I**) Venn-diagram of differentially expressed osteolytic, osteoblastic and breast cancer mediators in conditioned medium from the experiment described in panel G. **p < 0.01.

Breast cancer cells contribute to osteolysis through secretion of pro-inflammatory factors^4^. To explore the role of TRAF2 in this process, we took advantage of an *in vivo* supracalvarial injection and *ex-vivo* calvarial osteoblast organ models to assess osteolysis in response to MDA-231-BT conditioned medium in adult immuno-competent mice (unlike the MDA-231-BT nude mouse model described above) (Fig. 3A,D). Conditioned medium from MDA-231-BT cells overexpressing TRAF2 (MDA-231-BT-T2^OE^) induced osteolytic bone damage in calvarial bone *in vivo* (Fig. 3B,C) and *ex vivo* (Fig. 3E,F) that is characterized by significant loss in bone volume (p < 0.01). Histomorphometric analysis of the calvarial bone from the organ culture showed that conditioned medium from TRAF2 overexpressing cells increased osteoclast number (Fig. 3G, left panel) without affecting the number of osteoblasts (Fig. 3G, right panel). Next, we employed a quantitative proteomic approach to identify the tumour-derived factor(s) responsible. Analysis of protein level of human cytokines and chemokines in conditioned medium revealed that TRAF2 overexpression in the osteotropic MDA-231-BT-T2^OE^ is associated with upregulation of a total of 48 secreted proteins in the conditioned medium (Fig. 3G). The identified proteins are common tumour-derived factors that have previously been found to be involved in the regulation of inflammation, angiogenesis, innate immunity and tumorigenesis (Fig. 3G and Table S1). Further evaluation of the role of these proteins in breast cancer, osteoclastogenesis and/or osteoblast differentiation revealed a subset of 21 proteins that are likely to be implicated in the regulation of the TRAF2-driven breast cancer-induced osteoclast and osteoblast changes that we have observed in our models (Fig. 3H and Table S2).

### Cancer-specific TRAF2 regulates osteoblast and osteoclast changes associated with breast cancer

**Figure 4:**
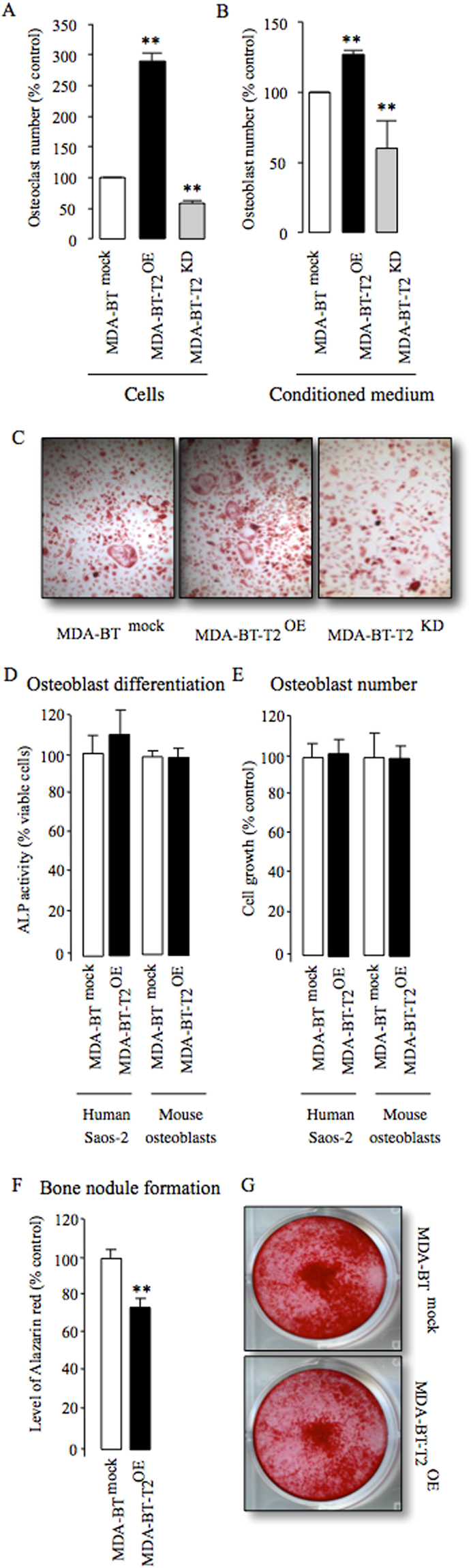
TRAF2 enhances breast cancer cell-induced bone cell activity. (**A**,**B**) *In vitro* osteoclastogenesis of murine M-CSF and RANKL stimulated bone marrow co-cultured with mock (MDA-BT ^mock^) control, TRAF2 overexpressing (MDA-BT-T2^OE^) and TRAF2 deficient (MDA-BT-T2^KD^) human breast cancer cells (**A**) or after exposure to conditioned medium (20% v/v) from these cells (**B**). (**C**) Representative photomicrographs of TRAcP positive multi-nucleated osteoclasts from the experiment described in A. (**D**,**E**) *In vitro* osteoblast differentiation (**D**) and viability (**E**) in mouse calvarial osteoblasts and the human osteoblast-like cells Saos-2 after exposure to conditioned medium (20% v/v) from mock (MDA-BT ^mock^) control or TRAF2 overexpressing MDA-BT-T2^OE^ human breast cancer cells for (2 days, calvarial osteoblasts, right) and (12 days, Saos-2, left) as assessed by Alkaline phosphatase (ALP) activity and Alamar Blue, respectively. (**F**) *In vitro* osteoblast bone nodule formation in cultures of the human osteoblast-like Saos-2 cells after exposure to conditioned medium (20% v/v) from mock (MDA-BT ^mock^) control or TRAF2 overexpressing MDA-BT-T2^OE^) human breast cancer cells as assessed by Alazarin Red. (**G**) Representative photomicrographs of bone nodule in the experiment described in F. **p < 0.01.

Previous studies have shown that TRAF2 expression in bone cells plays an important role in osteoclast differentiation and osteoblast apoptosis. Here, we carried out *in vitro* functional studies to examine the effects of knockdown and overexpression of cancer-specific TRAF2 on osteoclast and osteoblast changes associated with breast cancer. These experiments showed that TRAF2 overexpression significantly increased the ability of osteotropic MDA-231-BT cells (Fig. 4A) and their conditioned medium (20% v/v, Fig. 4B) to enhance RANKL-induced osteoclast formation in bone marrow cultures, whereas TRAF2 knockdown in these cells was inhibitory (Fig. 4A–C). Exposure of mouse primary osteoblasts and the human osteoblast-like cells Saos-2 cells to conditioned medium from the osteotropic MDA-231-BT cells overexpressing TRAF2 for 2 (mouse osteoblasts) or 12 (human Saos-2) days had no effect on osteoblast differentiation (Fig. 4D) or growth (Fig. 4E) *in vitro*. However, a modest yet significant reduction in bone nodule formation was detected in human Saos-2 cultures after 12 days of continuous exposure to conditioned medium from the osteotropic MDA-231-BT cells overexpressing TRAF2 when compared to control (Fig. 4F,G).

### TRAF2 regulates the behaviour of osteotropic breast cancer cells by engaging IKKβ and IKKε

**Figure 5:**
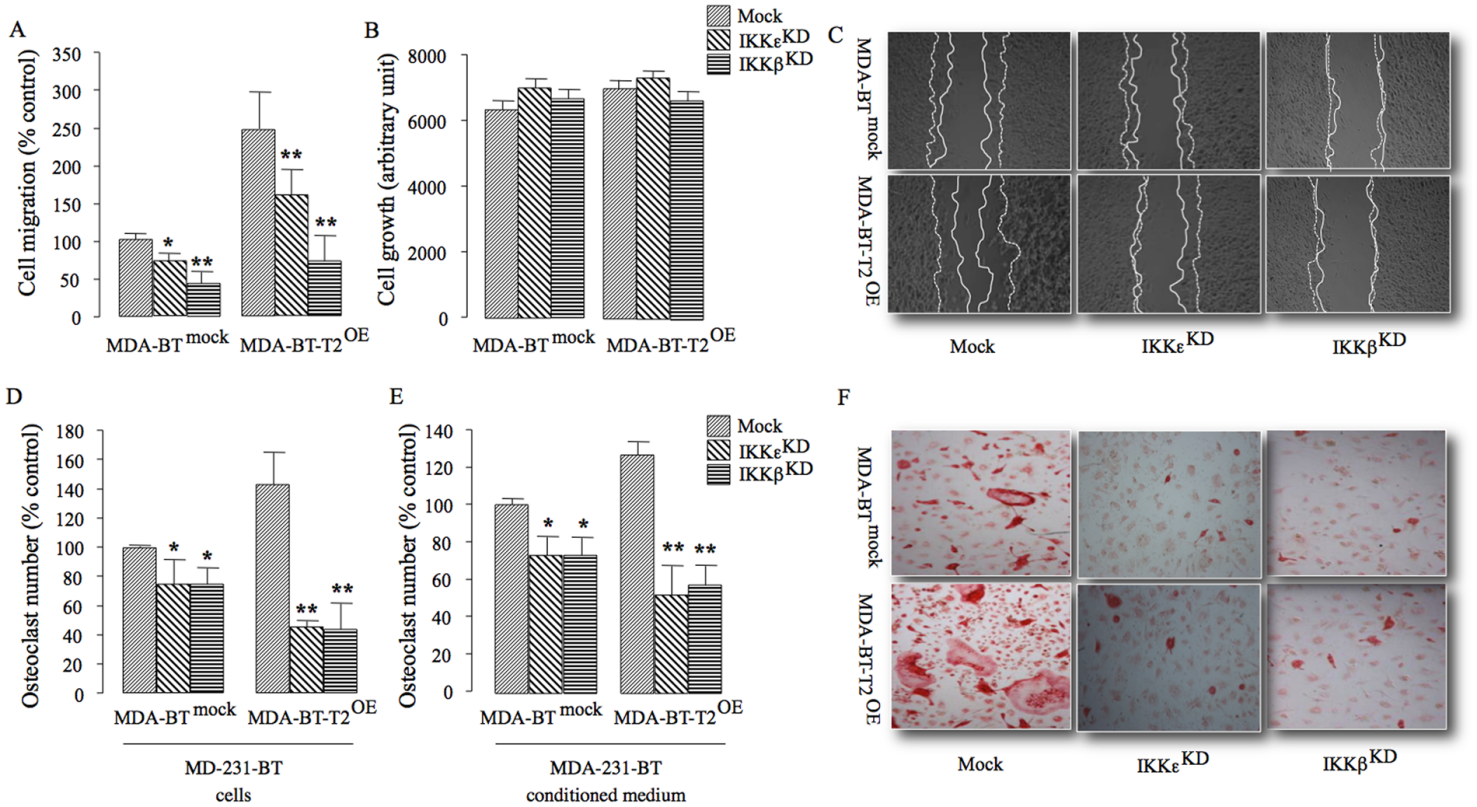
TRAF2 regulates osteolytic behaviour of osteotropic MDA-231-BT cells by engaging IKKβ and IKKε. (**A**) *In vitro* cell migration of the osteotropic human MDA-231-BT overexpressing TRAF2 (MDA-BT-T2^OE^) cells or their control (MDA-BT ^mock^) transfected with control (mock), IKKε (IKKε^ΚD^) or IKKβ (IKKβ^ΚD^) siRNA. (**B**) *In vitro* cell growth of the osteotropic human MDA-BT-T2^OE^ cells or their control MDA-BT ^mock^ from the experiment described in panel A. (**c**) Representative photomicrographs from the experiment described in panels A-B. (**D**,**E**) *In vitro* osteoclastogenesis of murine M-CSF and RANKL stimulated bone marrow after exposure to the osteotropic human MDA-231-BT overexpressing TRAF2 (MDA-BT-T2^OE^) cells or their control (MDA-BT^mock^) transfected with control (mock), IKKε (IKKε^ΚD^) or IKKβ (IKKβ^ΚD^) siRNA (**D**) or conditioned medium (20% v/v, e) from these cells. (**F**) Representative photomicrographs of TRAcP positive multi-nucleated osteoclasts from the experiment described in panel E. *p < 0.05, **p < 0.01.

Previous studies have shown that TRAF2-driven NFκB activation in breast cancer cells is primarily mediated by the expression and kinase activity of IKKβ and IKKε^3,6,7,29^. In view of this, we knocked down IKKβ and IKKε in the osteotropic MDA-231-BT cells overexpressing TRAF2 (Fig. S2) and we investigated the effects of these changes on these cells metastatic and osteolytic behaviour *in vitro*. As shown in Fig. 5, knockdown of IKKβ or IKKε in TRAF2 overexpressing osteotropic MDA-231-BT cells significantly inhibited cell migration (Fig. 5A and C) and reduced the ability of these cells to enhance RANKL-induced osteoclast formation in bone marrow cultures (Fig. 5D–F). Knockdown of IKKβ or IKKε expression had no effect on the growth of MDA-231-BT cells overexpressing TRAF2 (Fig. 5B).

## Discussion

TRAF2 has diverse functions in inflammation^3,29,30^, and its upregulation in mammary epithelial cells enhances their oncogenic transformation and promotes tumour growth^7^. In addition, TRAF2, through its interaction with NFκB, is implicated in the regulation of bone cell differentiation and bone remodelling^2,4,25–27,31–33^. Nonetheless, the contribution of TRAF2 to bone metastasis, skeletal tumour growth and osteolysis associated with advanced breast cancer has not been investigated. Our present *in vivo*, *ex vivo* and *in vitro* investigation showed that TRAF2 overexpression in parental and osteotropic sub-clones of MDA-231 human breast cancer cells increased tumour cell growth and motility, whereas its knockdown is inhibitory. TRAF2 overexpression also enhanced the ability of osteotropic breast cancer cells and their derived factors to induce osteoclast formation, inhibit osteoblast differentiation and to cause osteolysis by a mechanism that depends at least in part on NFκB activation.

Malignant breast cancer cells accumulate oncogenic alterations that significantly affect their growth in the primary site and metastatic activity at distant sites such as bone^34,35^. The majority of studies thus far implicate the TRAF2/NFκB pathway in these processes were limited to the role of TRAF2 in mammary tumour growth^6,10,12,17,19,21–23^. Here, and in broad agreement with these reports, we have found that TRAF2 overexpression promoted both mammary and skeletal tumour growth in mice after orthotopic and intra-tibial injections. NFκB signalling in breast cancer cells facilitates tumour growth and spread through induction of inflammatory mediators^36^. *In vitro* examination of the metastatic behavior of parental and osteotropic cells confirmed that TRAF2 overexpression is linked to increased tumour cell motility and invasion, whereas knockdown of TRAF2 was inhibitory. Examination of the levels of secreted proteins in conditioned medium from these cultures revealed that TRAF2 overexpression in osteotropic cells is associated with elevated levels of various pro-tumour and pro-migratory factors including uPAR, VEGF, IL-1, TSP-1 and Serpine 1, each with functions that enable primary and metastatic breast cancer cells to spread (Table S1).

Bone metastasis in advanced breast cancer is predominantly osteolytic^37^. Malignant breast cancer cells in bone acquire new characteristics influenced by matrix-derived factors^24,37–40^. In the present study, we provide evidence to support the notion that cancer-specific TRAF2 contributes to breast cancer cell behavior in bone. The evidence for this comes from the experiments that showed that (A) TRAF2 is highly expressed in the osteotropic MDA-231-BT cells when compared to parental control, (B) knockdown of TRAF2 in osteotropic MDA-231-BT inhibited cell migration and reduced the ability of these cells to enhance osteoclastogenesis, (C) conditioned medium from osteotropic MDA-231-BT overexpressing TRAF2 reduced osteoblast ability to form bone nodules *in vitro*, and (D) upregulation of TRAF2 worsened breast cancer-induced osteolysis. Microarray analysis of secreted proteins in conditioned medium from osteotropic MDA-231-BT overexpressing TRAF2 indicates that these effects were likely due to increase in the level of tumour-derived NFκB-mediated osteolytic factors, such as TNFα, IL-1, granulate macrophage colony stimulating factor (GM-CSF), M-CSF, CD40L, IL-17, IL-11, vascular endothelial growth factor (VEGF) (Table S1). TRAF2 overexpression also enhanced the level of tumour-derived TNFα, a known an inhibitory factor of osteoblast survival and differentiation^4,25,33^. Thus, it is reasonable to speculate that TRAF2 in osteotropic breast cancer cells orchestrates the expression of a set of tumour-derived factors that function cooperatively to alter the balance between osteoblasts and osteoclasts. Nevertheless, it is important to note that we cannot exclude the role of well-established systemic and bone-derived osteolytic mediators such as RANKL, insulin growth factor 1 (IGF-1) and parathyroid hormone-related protein (PTHrP) in TRAF2-induced osteolysis in our models.

A number of studies have shown that TRAF2 oncogenic activity in parental breast cancer cells is driven by IKKβ and IKKε^3,6,7,29,41^. In the skeleton, selective inhibition of IKKε or IKKβ have been found to inhibit osteoclastic bone resorption^28,42–44^ and promote bone formation^45,46^. Here, we provided further evidence that IKKβ and IKKε are also implicated in TRAF2-driven metastatic and osteolytic behavior of the osteotropic MDA-231-BT cells. To this end, we showed that selective silencing of either IKKβ or IKKε reduced the ability of TRAF2 overexpressing MDA-231-BT cells to migrate and to enhance RANKL-induced osteoclastogenesis. Altogether, these results demonstrate that TRAF2 contributes to breast cancer related osteolysis through its regulation of both the canonical and non-canonical NFκB signalling pathway.

In conclusion, our studies showed that the TRAF2/IKK/NFκB axis in osteotropic breast cancer cells contributes to breast cancer associated osteolytic bone damage. We also confirmed that cancer-specific expression of TRAF2 regulates mammary tumour growth and showed for the first time that TRAF2 is implicated in skeletal tumour burden. When combined with previous studies that demonstrated that TRAF2 plays an important role in breast cancer initiation^3,6,7,29^, our present findings indicate that targeting of TRAF2 may have a potential therapeutic value in halting breast cancer metastasis and protecting the skeleton from the osteolysis associated with advanced breast cancer.

## Methods

### Materials and reagents

The parental human MDA-MB-231 (MDA-231) as representative of triple-negative breast cancer cells and human osteoblast-like cells Saos-2 were purchased from ATCC (Manassas, VA). The osteotropic MDA-231-BT1 was generated by repeated passages *in vivo* and validated for their ability to colonize bone and to cause osteolysis^47^. Tissue culture medium (DMEM and alpha-MEM) was obtained Sigma-Aldrich (Dorset, UK). All primary antibodies were purchased from Cell Signalling Biotechnology (MA, USA) except rabbit anti-actin was obtained from Sigma-Aldrich (Dorset, UK). Mouse macrophage colony stimulating factor (M-CSF) was obtained from R&D Systems (Abingdon, UK) and receptor activator of NFκB ligand (RANKL) was a gift from Patrick Mollat (Galapagos SASU, France).

### Generation of stable cell lines

TRAF2 was overexpressed in human parental MDA-231 and osteotropic MDA-231-BT breast cancer cells using the retroviral gene delivery system. The TRAF2 constructs used were a gift from the Wilkinson Lab at the Edinburgh Cancer Research UK Centre (University of Edinburgh). The Gateway donor vector TRAF2 from pDONR223 was recombined into Gateway destination vector MSCV-N-Flag-HA-IRES-PURO by LTR recombination (Behrends *et al*., 2010). MSCV-N-Flag-HA-IRES-PURO vector alone (Empty vector-MOCK) was used as the control. EGFP-MSCV-N-Flag-HA-IRES-PURO construct (EGFP) was used as a control to determine the transfection efficiency. HEK293ET cells were exposed to transfection mixture (5 μg of vector DNA, 5 μg of gag and pol, 5 μg of pMD2.G, 40 μl of Polyethylenimine (PEI) transfection reagent and 450 μl of standard DMEM supplemented with 10% FCS). MDA-231 and MDA-231-BT cells were infected using an infection mixture containing 14 μl of Polybrene transfection reagent, 5 ml of harvested virus and 9 ml of medium was prepared and filtered (Percentage of virus 5/14*100 = 35.7%). EGFP construct transfected cells were used to estimate the transfection efficiency.

### Small RNA interference

Parental and osteotropic MDA-231 cells were transfected with siRNA (25 nM) using Dharmafect 1 reagent (Dharmacon, CO, USA), according to the manufacturer’s instructions. Small interfering RNAs (siRNA; siGenome SMART pool) as a pool of four annealed double-stranded RNA oligonucleotides for IKKβ (M-003503-03), IKKε (M-003723-02), TRAF2 (M-005198-00-0005), and non-targeting control no. 3 (D-001210-03-05) were used according to the manufacturer’s instructions. For transfections involving the combination of TRAF2 and IKKβ oligonucleotide, each oligonucleotide was transfected at a concentration of 50 nM, so the total siRNA concentration is 100 nM. The cells were cultured for 48 hours in antibiotic free complete medium with the transfection reagent. The efficiency of TRAF2, IKKε and IKKβ knockdown and overexpression was assessed by Western blot analysis.

### Animal experiments

All experimental protocols were approved by the Ethics Committee at the University of Edinburgh (Scotland, United Kingdom) and L’Aquila (Italy) and were conducted in accordance with the United Kingdom Home Office regulations.

### Intra-tibial injection in mice

The effect of TRAF2 overexpression on skeletal tumour growth and local osteolysis was studied by intra-tibial injection of osteotropic MDA-MB-231 (MDA-231-BT) breast cancer cell-line in 4-week-old female Balb/c nu/nu mice. Briefly, mice were divided into two groups and received intra-tibial injection of human breast cancer cells mock (MDA-231-BT ^mock^) or cells overexpressing TRAF2 (MDA-231-BT-T2^OE^) (5 × 10^4^ cells) in the left leg or a sham injection of Phosphate buffered saline (PBS) into the right leg. Animals were euthanized 14 days post injection and bones were analyzed by micro–computed tomography (microCT, Skyscan 1172 scanner)^48^. Skeletal tumor growth was measured on 2D microCT images using Brucker ctAN and Image J (1.34 s; NIH, Bethesda, MD, USA) and results were expressed as a percentage of total metaphyseal area.

### Supracalvarial injection in mice

The effects of TRAF2 overexpression on breast cancer-induced osteolysis was studied by supracalvarial injection of conditioned medium in immuno-competent 3-week-old C57Bl/6 female mice. Mice were injected subcutaneously over the calvarial bones with conditioned medium (50μL) from human mock (MDA-231-BT ^mock^) or cells overexpressing TRAF2 (MDA-231-BT-T2^OE^) breast cancer cells on 5 consecutive days. The calvarial bone was assessed using microCT at a resolution of 8μm^48^.

### Bone organ culture system

*Ex vivo* osteolysis was studied in mouse calvarial bone using an adaptation of the mouse calvarial organ culture^49^. Mouse calvarias were isolated from 7-day-old mice, divided into equal halves and cultured on stainless steel rafts in MDA-BT-mock or MDA-BT-T2OE conditioned medium (20% v/v). Tissue culture medium was refreshed every 48 hours and the cultures were terminated after 7 days. Bone volume was assessed by microCT (resolution, 5μm) as previously described^48^. Bone histomorphometry was performed on the calvarial bone and Hematoxilin and Eosin staining was used to evaluate osteoclasts and toluidine blue staining for osteoblasts. Histological sections were analysed using software based on the Aphelion Image Analysis tool kit (Adcis, He0rouville- Saint-Clair, France).

### Osteoblast cultures

Primary osteoblasts were isolated from the calvarial bones of 2-day-old mice^50^. Primary calvarial osteoblasts (15 × 10^3^ cells/well) and the osteoblast-like cells Saos-2 (10 × 10^5^ cells/well) were cultured in standard alpha-MEM or DMEM respectively, supplemented β-glycerol phosphate (10 μM) and L-ascorbic acid (50 µg/ml) for 2 (osteoblast differentiation), 12 (bone nodule, Saos-2) or 21 (bone nodule, calvarial osteoblasts) days. Osteoblast cell number, differentiation and bone nodule formation were determined by AlamarBlue assay, alkaline phosphatase (ALP) assay and alizarin red (ALZ) staining^50^.

### Osteoclast cultures

Osteoclast formation was studied in RANKL and M-CSF stimulated bone marrow cultures. Bone marrow (BM) cells were obtained from the marrow of 3-month-old mice and M-CSF-dependent osteoclast precursor cells generated as previously described^51^. M-CSF-dependent osteoclast precursors were plated into 96 well plates (15 × 10^3^ cells/well) in alpha-MEM supplemented with M-CSF (25 ng/ml) and RANKL (100 ng/ml) for 6 hours prior addition of MDA-231 breast cancer cells (300 cells/well) or their conditioned medium (20% v/v). Cultures were stained for Tartrate-Resistant Acid Phosphatase (TRAcP)^50^.

### Western Blotting

Western blot analysis was used to detect protein expression in breast cancer cells. Cells were lysed in a standard buffer (0.1% (w/v) Sodium dodecyl sulfate, 0.5% (w/v) sodium deoxycholate, 1% Triton X-100, 1 μM Ethylenediaminetetraacetic acid, 2% (v/v) protease inhibitor cocktail, 10 µM of sodium fluoride and 2% (v/v) phosphatase inhibitor cocktail). Protein concentration was determined using BCA assay (Pierce, USA). Total protein lysate (60μg) was resolved by SDS-PAGE (BioRAD, United Kingdom), immunoblotted with antibodies according to manufacturer’s instructions, detected using rabbit monoclonal antibodies (all at 1:1000 dilution, cell Signalling Technology, USA) and immuno-complexes were visualised by enhanced chemiluminescence (Amersham, UK) on a Syngene GeneGnome imaging system. The intensity of the bands was quantified using GeneSnap software (Syngene, UK) and level of actin was used for normalization.

### Measurement of levels of tumour-derived factors

Level of tumour-derived factors in conditioned medium from human MDA-231 breast cancer cells was determined by Proteome Profiler Human XL Cytokine Array Kit (ARY022, R&D Systems, Abingdon, UK), according to the manufacturer’s instructions.

### Statistical analysis

Comparison between groups was done by analysis of variance (ANOVA) followed by Dunnet’s post hoc test (SPSS for Windows, version 11). A p-value value of 0.05 or below was considered statistically significant.

## Electronic supplementary material

Supplementary materials
