# Supplementary material for: TRAF2 in osteotropic breast cancer cells enhances skeletal tumour growth and promotes osteolysis
Source: Sci Rep. 2018 Jan 8;8:39. doi: 10.1038/s41598-017-18327-5 (PMC5758572; doi:10.1038/s41598-017-18327-5)
Supplement: Supplementary file 1 — Supplementary materials [file 41598_2017_18327_MOESM1_ESM.doc]

**TRAF2 in osteotropic breast cancer cells enhances skeletal tumour growth and promotes osteolysis**

**Running title**

TRAF2 enhances breast cancer-induced osteolysis.

**Author affiliations**

Prabha Peramuhendige^1,2^, Silvia Marino^1,2^, Ryan T. Bishop^1^, Daniëlle de Ridder^1^, Asim Khogeer^1,2^, Isabella Baldini^3^, Mattia Capulli^3^, Nadia Rucci^3^ and Aymen I. Idris^1,2^

^1^Department of Oncology and Metabolism, University of Sheffield, Medical School, Beech Hill Road, Sheffield, S10 2RX, UK.

^2^Bone and Cancer Group, Edinburgh Cancer Research Centre, MRC Institute of Genetics and Molecular Medicine, University of Edinburgh, EH4 2XR, UK.

^3^University of L’Aquila, Department of Biotechnological and Applied Clinical Sciences, L’Aquila, Italy.

**Contact**

Dr Aymen I. Idris. Department of Oncology and Metabolism, Medical School, Beech Hill Road, Sheffield, S10 2RX, UK. E-mail: aymen.idris@sheffield.ac.uk.

**Supplementary materials**

**FIGURES**

**Figure S1.**

**
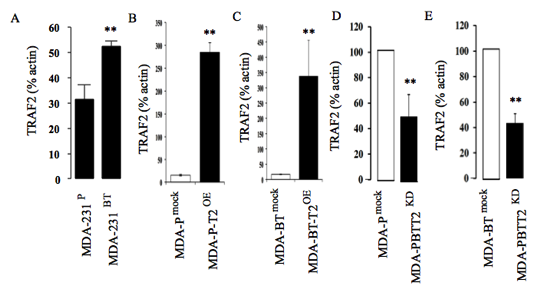
**

**Figure S1 (***related to Figure 1***). Expression of TRAF2 in parental and osteotropic sub-clones of MDA-231 cell lines.** (A) Protein expression of TRAF2 in parental (MDA-231-P) and osteotropic (MDA-231-BT) human MDA-231 breast cancer cells. (B-C) Protein expression of TRAF2 in parental (B) and osteotropic (C) human MDA-231 breast cancer cells stably transduced with retroviral vector for TRAF2 or mock control. (D-E) Protein expression of TRAF2 in parental (D) and osteotropic (E) human MDA-231 breast cancer cells stably transduced with shRNA (25nmol/l) (Dharmafect, UK) for TRAF2 or mock control. Protein probed with rabbit anti-TRAF2 and rabbit anti-actin. Values in the graphs are mean ± S.D. and are obtained from 3 independent experiments. **p < 0.001 from mock control. Abbreviation: M-molecular weight marker (KDa).

**Figure S2.**

**
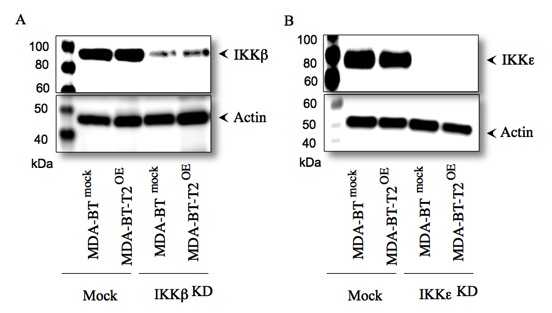
**

**Figure S2 (***related to Figure 5***). Successful knockdown of IKKβ and IKKε in the osteotropic MDA-231-BT cells.** Protein expression and Western blot of IKKβ (A) and IKKε (B) in osteotropic human MDA-231 breast cancer cells transfected with IKKβ or IKKε siRNA (25nmol/l) with Dharmafect 1 reagent according to the manufacturer’s instructions. Protein probed with rabbit anti-IKKβ, anti-IKKε and rabbit anti-actin.

**TABLES**

**Table S1** (related to Figure 3, panel G). Levels of human cytokines and chemokines in conditioned medium from the osteotropic MDA-231-BT breast cancer cell line as measured by Proteome Profiler Human XL Cytokine Array Kit. Abbreviation; Ref, references.

| **Protein** | **Breast cancer** | **Bone metastasis** | **Osteoclast** | **Osteoblast** | **Ref** |
| --- | --- | --- | --- | --- | --- |
| Endoglin | ✓ |  |  | ✓ | 1, 2 |
| Vascular endothelial growth factor (VEGF) | ✓ | ✓ | ✓ | ✓ | 3, 4 |
| Angiogenin (ANG) | ✓ |  | ✓ |  | 5, 6 |
| Angiopoietin-1 (ANGPT1) | ✓ | ✓ | ✓ | ✓ | 7, 8 |
| Angiopoietin-2 (ANGPT2) | ✓ | ✓ |  | ✓ | 9, 10 |
| Factor-D (CFD) | ✓ |  |  |  | 11 |
| Complement protein C (C5) | ✓ |  |  |  | 12 |
| CD40 ligand | ✓ |  | ✓ | ✓ | 13-15 |
| Interferon gamma (IFNγ) | ✓ | ✓ | ✓ | ✓ | 16-18 |
| Cluster of differentiation 14 (CD14) | ✓ | ✓ | ✓ |  | 19, 20 |
| CD147 | ✓ | ✓ | ✓ |  | 21 |
| Trefoil factor 3 | ✓ | ✓ |  |  | 22 |
| Fibroblast growth factor 2 (FGF-2) | ✓ | ✓ | ✓ | ✓ | 23, 24 |
| Epidermal growth factor (EGF) | ✓ | ✓ | ✓ | ✓ | 25, 26 |
| FGF19 | ✓ |  |  | ✓ | 27, 28 |
| Urokinase receptor (uPAR) | ✓ | ✓ | ✓ | ✓ | 29 |
| SERPINE1 | ✓ | ✓ |  |  | 30 |
| Dickkopf WNT inhibitor 1 (DKK1) | ✓ | ✓ | ✓ | ✓ | 31, 32 |
| Thrombospondin-1 (TSP1) | ✓ |  |  |  | 33 |
| Pentraxin-related protein 3 (PTX3) | ✓ | ✓ | ✓ | ✓ | 34, 35 |
| CXC Motif Chemokine Ligand 5 (CXCL5) | ✓ | ✓ |  | ✓ | 36-38 |
| Chemokine (C-C motif) ligand (CCL17) | ✓ |  |  |  | 39 |
| CCL8 | ✓ |  |  |  | 40 |
| CXCL11 | ✓ | ✓ |  | ✓ | 41 |
| CCL20 | ✓ | ✓ | ✓ | ✓ | 42-44 |
| CCl19 | ✓ | ✓ | ✓ |  | 41, 45 |
| CXCL10 | ✓ | ✓ | ✓ |  | 46 |
| CXCL1 | ✓ | ✓ | ✓ | ✓ | 47, 48 |
| CCL2 | ✓ | ✓ | ✓ | ✓ | 49, 50 |
| Interleukin 4 (IL4) | ✓ |  | ✓ | ✓ | 51 |
| IL11 | ✓ | ✓ | ✓ | ✓ | 52-54 |
| IL17 | ✓ | ✓ | ✓ | ✓ | 55-57 |
| Granulate colony stimulating factor (G-CSF) | ✓ | ✓ | ✓ | ✓ | 58 |
| IL-10 | ✓ |  | ✓ | ✓ | 59, 60 |
| sST2 | ✓ |  |  |  | 59, 61 |
| IL-22 | ✓ |  | ✓ |  | 62, 63 |
| IL-1 | ✓ | ✓ | ✓ | ✓ | 64-66 |
| TNFα | ✓ | ✓ | ✓ | ✓ | 67, 68 |
| Macrophage migration inhibitory factor (MIF) | ✓ |  | ✓ |  | 69, 70 |
| IL33 | ✓ |  | ✓ |  | 71, 72 |
| IL8 | ✓ | ✓ | ✓ |  | 73 |
| M-CSF | ✓ | ✓ | ✓ |  | 74, 75 |
| IL-27 | ✓ |  | ✓ | ✓ | 76-78 |
| Lipocalin-2 (LCN2) | ✓ |  | ✓ | ✓ | 79-81 |
| GM-CSF | ✓ | ✓ | ✓ | ✓ | 82, 83 |
| C-reactive protein (CRP) | ✓ | ✓ | ✓ |  | 84-86 |
| FLT3 ligand | ✓ | ✓ | ✓ |  | 87, 88 |
| Transforming Growth Factor Alpha (TGFα) | ✓ | ✓ | ✓ | ✓ | 89-91 |

**Table S2 (**related to figure 3, panel H**).** Differential expression of osteolytic, osteoblastic and breast cancer mediators in conditioned medium from the osteotropic MDA-231-BT breast cancer cell line. Refer to table 1 for abbreviation and references.

| Breast cancer | Breast cancer, Osteoclasts, Osteoblasts | Breast cancer,  Osteoclasts | Breast cancer Osteoblasts |
| --- | --- | --- | --- |
| C5  CCL17  CCl8  CD40L  CFD  CXCL11  SERPINE1  TFF3  TSP1  sST2 | VEGF  ANGPT1  IFNG  FGF2  EGF  uPAR  DKK1  PTX3  CCL20  CXCL1  CCL2  IL4  IL11  IL17  G-CSF  IL10  IL-1  TNFA  IL27  LCN2  GMCSF  TGFα | ANG  CCl19  CD14  CD147  CRP  CXCL10  FLT3LG  IL22  IL33  IL8  MCSF  MIF | ANGPT2  CXCL5  ENG  FGF19 |

**REFRENCES** (related to Tables 1 and 2)

1 Ishibashi O, Inui T. Identification of endoglin-dependent BMP-2-induced genes in the murine periodontal ligament cell line PDL-L2. J Mol Signal 2014; 9: 5.

2 Oxmann D, Held-Feindt J, Stark AM, Hattermann K, Yoneda T, Mentlein R. Endoglin expression in metastatic breast cancer cells enhances their invasive phenotype. Oncogene 2008; 27: 3567-3575.

3 Hu K, Olsen BR. Osteoblast-derived VEGF regulates osteoblast differentiation and bone formation during bone repair. The Journal of clinical investigation 2016; 126: 509-526.

4 Aldridge SE, Lennard TW, Williams JR, Birch MA. Vascular endothelial growth factor receptors in osteoclast differentiation and function. Biochem Biophys Res Commun 2005; 335: 793-798.

5 Sasaki A, Alcalde RE, Nishiyama A, Lim DD, Mese H, Akedo H *et al*. Angiogenesis inhibitor TNP-470 inhibits human breast cancer osteolytic bone metastasis in nude mice through the reduction of bone resorption. Cancer Res 1998; 58: 462-467.

6 Montero S, Guzman C, Cortes-Funes H, Colomer R. Angiogenin expression and prognosis in primary breast carcinoma. Clinical cancer research : an official journal of the American Association for Cancer Research 1998; 4: 2161-2168.

7 Suzuki T, Miyamoto T, Fujita N, Ninomiya K, Iwasaki R, Toyama Y *et al*. Osteoblast-specific Angiopoietin 1 overexpression increases bone mass. Biochem Biophys Res Commun 2007; 362: 1019-1025.

8 Xie C, Schwarz EM, Sampson ER, Dhillon RS, Li D, O'Keefe RJ *et al*. Unique angiogenic and vasculogenic properties of renal cell carcinoma in a xenograft model of bone metastasis are associated with high levels of vegf-a and decreased ang-1 expression. J Orthop Res 2012; 30: 325-333.

9 Takano A, Fukuda T, Shinjo T, Iwashita M, Matsuzaki E, Yamamichi K *et al*. Angiopoietin-like protein 2 is a positive regulator of osteoblast differentiation. Metabolism 2017; 69: 157-170.

10 Han HH, Kim BG, Lee JH, Kang S, Kim JE, Cho NH. Angiopoietin-2 promotes ER+ breast cancer cell survival in bone marrow niche. Endocrine-related cancer 2016; 23: 609-623.

11 Zheng ZH, Yang Y, Lu XH, Zhang H, Shui XX, Liu C *et al*. Mycophenolic acid induces adipocyte-like differentiation and reversal of malignancy of breast cancer cells partly through PPARgamma. European journal of pharmacology 2011; 658: 1-8.

12 Gunn L, Ding C, Liu M, Ma Y, Qi C, Cai Y *et al*. Opposing roles for complement component C5a in tumor progression and the tumor microenvironment. J Immunol 2012; 189: 2985-2994.

13 Tong AW, Papayoti MH, Netto G, Armstrong DT, Ordonez G, Lawson JM *et al*. Growth-inhibitory effects of CD40 ligand (CD154) and its endogenous expression in human breast cancer. Clinical cancer research : an official journal of the American Association for Cancer Research 2001; 7: 691-703.

14 Lomaga MA, Yeh WC, Sarosi I, Duncan GS, Furlonger C, Ho A *et al*. TRAF6 deficiency results in osteopetrosis and defective interleukin-1, CD40, and LPS signaling. Genes Dev 1999; 13: 1015-1024.

15 Robinson JW, Li JY, Walker LD, Tyagi AM, Reott MA, Yu M *et al*. T cell-expressed CD40L potentiates the bone anabolic activity of intermittent PTH treatment. Journal of bone and mineral research : the official journal of the American Society for Bone and Mineral Research 2015; 30: 695-705.

16 Duque G, Huang DC, Macoritto M, Rivas D, Yang XF, Ste-Marie LG *et al*. Autocrine regulation of interferon gamma in mesenchymal stem cells plays a role in early osteoblastogenesis. Stem Cells 2009; 27: 550-558.

17 Garcia-Tunon I, Ricote M, Ruiz AA, Fraile B, Paniagua R, Royuela M. Influence of IFN-gamma and its receptors in human breast cancer. BMC Cancer 2007; 7: 158.

18 Xu Z, Hurchla MA, Deng H, Uluckan O, Bu F, Berdy A *et al*. Interferon-gamma targets cancer cells and osteoclasts to prevent tumor-associated bone loss and bone metastases. The Journal of biological chemistry 2009; 284: 4658-4666.

19 Taylor RM, Kashima TG, Hemingway FK, Dongre A, Knowles HJ, Athanasou NA. CD14- mononuclear stromal cells support (CD14+) monocyte-osteoclast differentiation in aneurysmal bone cyst. Laboratory investigation; a journal of technical methods and pathology 2012; 92: 600-605.

20 Kyrgidis A, Yavropoulou MP, Lagoudaki R, Andreadis C, Antoniades K, Kouvelas D. Increased CD14+ and decreased CD14- populations of monocytes 48 h after zolendronic acid infusion in breast cancer patients. Osteoporos Int 2017; 28: 991-999.

21 Rucci N, Millimaggi D, Mari M, Del Fattore A, Bologna M, Teti A *et al*. Receptor activator of NF-kappaB ligand enhances breast cancer-induced osteolytic lesions through upregulation of extracellular matrix metalloproteinase inducer/CD147. Cancer Res 2010; 70: 6150-6160.

22 Pandey V, Wu ZS, Zhang M, Li R, Zhang J, Zhu T *et al*. Trefoil factor 3 promotes metastatic seeding and predicts poor survival outcome of patients with mammary carcinoma. Breast cancer research : BCR 2014; 16: 429.

23 Fei Y, Gronowicz G, Hurley MM. Fibroblast growth factor-2, bone homeostasis and fracture repair. Curr Pharm Des 2013; 19: 3354-3363.

24 Brady N, Chuntova P, Bade LK, Schwertfeger KL. The FGF/FGFR axis as a therapeutic target in breast cancer. Expert Rev Endocrinol Metab 2013; 8: 391-402.

25 Yi T, Lee HL, Cha JH, Ko SI, Kim HJ, Shin HI *et al*. Epidermal growth factor receptor regulates osteoclast differentiation and survival through cross-talking with RANK signaling. J Cell Physiol 2008; 217: 409-422.

26 Lu X, Kang Y. Epidermal growth factor signalling and bone metastasis. Br J Cancer 2010; 102: 457-461.

27 Lee YC, Gajdosik MS, Josic D, Clifton JG, Logothetis C, Yu-Lee LY *et al*. Secretome analysis of an osteogenic prostate tumor identifies complex signaling networks mediating cross-talk of cancer and stromal cells within the tumor microenvironment. Mol Cell Proteomics 2015; 14: 471-483.

28 Buhmeida A, Dallol A, Merdad A, Al-Maghrabi J, Gari MA, Abu-Elmagd MM *et al*. High fibroblast growth factor 19 (FGF19) expression predicts worse prognosis in invasive ductal carcinoma of breast. Tumour Biol 2014; 35: 2817-2824.

29 Hao W, Friedman A. Serum uPAR as Biomarker in Breast Cancer Recurrence: A Mathematical Model. PloS one 2016; 11: e0153508.

30 ordi Temprana DS, Juan Morote, Santiago Ramon y Cajal, Mireia Olivan, Marta Garcia. SERPINE1 Is Associated with Prostate Cancer Bone Metastases. *NATURE ANNUAL MEETING ABSTRACTS*. Springer Nature, 2017, p 221A.

31 Qiang YW, Barlogie B, Rudikoff S, Shaughnessy JD, Jr. Dkk1-induced inhibition of Wnt signaling in osteoblast differentiation is an underlying mechanism of bone loss in multiple myeloma. Bone 2008; 42: 669-680.

32 Mariz K, Ingolf JB, Daniel H, Teresa NJ, Erich-Franz S. The Wnt inhibitor dickkopf-1: a link between breast cancer and bone metastases. Clinical & experimental metastasis 2015; 32: 857-866.

33 Yee KO, Connolly CM, Duquette M, Kazerounian S, Washington R, Lawler J. The effect of thrombospondin-1 on breast cancer metastasis. Breast cancer research and treatment 2009; 114: 85-96.

34 Lee EJ, Song DH, Kim YJ, Choi B, Chung YH, Kim SM *et al*. PTX3 stimulates osteoclastogenesis by increasing osteoblast RANKL production. J Cell Physiol 2014; 229: 1744-1752.

35 Choi B, Lee EJ, Song DH, Yoon SC, Chung YH, Jang Y *et al*. Elevated Pentraxin 3 in bone metastatic breast cancer is correlated with osteolytic function. Oncotarget 2014; 5: 481-492.

36 Dang H, Wu W, Wang B, Cui C, Niu J, Chen J *et al*. CXCL5 Plays a Promoting Role in Osteosarcoma Cell Migration and Invasion in Autocrine- and Paracrine-Dependent Manners. Oncol Res 2017; 25: 177-186.

37 Bersini S, Jeon JS, Dubini G, Arrigoni C, Chung S, Charest JL *et al*. A microfluidic 3D in vitro model for specificity of breast cancer metastasis to bone. Biomaterials 2014; 35: 2454-2461.

38 Hsu YL, Hou MF, Kuo PL, Huang YF, Tsai EM. Breast tumor-associated osteoblast-derived CXCL5 increases cancer progression by ERK/MSK1/Elk-1/snail signaling pathway. Oncogene 2013; 32: 4436-4447.

39 Li JY, Ou ZL, Yu SJ, Gu XL, Yang C, Chen AX *et al*. The chemokine receptor CCR4 promotes tumor growth and lung metastasis in breast cancer. Breast cancer research and treatment 2012; 131: 837-848.

40 Farmaki E, Chatzistamou I, Kaza V, Kiaris H. A CCL8 gradient drives breast cancer cell dissemination. Oncogene 2016; 35: 6309-6318.

41 Kim HJ, Park J, Lee SK, Kim KR, Park KK, Chung WY. Loss of RUNX3 expression promotes cancer-associated bone destruction by regulating CCL5, CCL19 and CXCL11 in non-small cell lung cancer. The Journal of pathology 2015; 237: 520-531.

42 Pathak JL, Bakker AD, Verschueren P, Lems WF, Luyten FP, Klein-Nulend J *et al*. CXCL8 and CCL20 Enhance Osteoclastogenesis via Modulation of Cytokine Production by Human Primary Osteoblasts. PloS one 2015; 10: e0131041.

43 D'Amico L, Belisario D, Migliardi G, Grange C, Bussolati B, D'Amelio P *et al*. C-met inhibition blocks bone metastasis development induced by renal cancer stem cells. Oncotarget 2016; 7: 45525-45537.

44 Marsigliante S, Vetrugno C, Muscella A. CCL20 induces migration and proliferation on breast epithelial cells. J Cell Physiol 2013; 228: 1873-1883.

45 Cassier PA, Treilleux I, Bachelot T, Ray-Coquard I, Bendriss-Vermare N, Menetrier-Caux C *et al*. Prognostic value of the expression of C-Chemokine Receptor 6 and 7 and their ligands in non-metastatic breast cancer. BMC Cancer 2011; 11: 213.

46 Jin WJ, Kim B, Kim D, Park Choo HY, Kim HH, Ha H *et al*. NF-kappaB signaling regulates cell-autonomous regulation of CXCL10 in breast cancer 4T1 cells. Exp Mol Med 2017; 49: e295.

47 Hardaway AL, Herroon MK, Rajagurubandara E, Podgorski I. Marrow adipocyte-derived CXCL1 and CXCL2 contribute to osteolysis in metastatic prostate cancer. Clinical & experimental metastasis 2015; 32: 353-368.

48 Acharyya S, Oskarsson T, Vanharanta S, Malladi S, Kim J, Morris PG *et al*. A CXCL1 paracrine network links cancer chemoresistance and metastasis. Cell 2012; 150: 165-178.

49 Mizutani K, Sud S, McGregor NA, Martinovski G, Rice BT, Craig MJ *et al*. The chemokine CCL2 increases prostate tumor growth and bone metastasis through macrophage and osteoclast recruitment. Neoplasia 2009; 11: 1235-1242.

50 Bonapace L, Coissieux MM, Wyckoff J, Mertz KD, Varga Z, Junt T *et al*. Cessation of CCL2 inhibition accelerates breast cancer metastasis by promoting angiogenesis. Nature 2014; 515: 130-133.

51 Riancho JA, Gonzalez-Marcias J, Amado JA, Olmos JM, Fernandez-Luna JL. Interleukin-4 as a bone regulatory factor: effects on murine osteoblast-like cells. J Endocrinol Invest 1995; 18: 174-179.

52 McCoy EM, Hong H, Pruitt HC, Feng X. IL-11 produced by breast cancer cells augments osteoclastogenesis by sustaining the pool of osteoclast progenitor cells. BMC Cancer 2013; 13: 16.

53 Suga K, Saitoh M, Fukushima S, Takahashi K, Nara H, Yasuda S *et al*. Interleukin-11 induces osteoblast differentiation and acts synergistically with bone morphogenetic protein-2 in C3H10T1/2 cells. J Interferon Cytokine Res 2001; 21: 695-707.

54 Ren L, Wang X, Dong Z, Liu J, Zhang S. Bone metastasis from breast cancer involves elevated IL-11 expression and the gp130/STAT3 pathway. Medical oncology 2013; 30: 634.

55 Croes M, Oner FC, van Neerven D, Sabir E, Kruyt MC, Blokhuis TJ *et al*. Proinflammatory T cells and IL-17 stimulate osteoblast differentiation. Bone 2016; 84: 262-270.

56 Yago T, Nanke Y, Ichikawa N, Kobashigawa T, Mogi M, Kamatani N *et al*. IL-17 induces osteoclastogenesis from human monocytes alone in the absence of osteoblasts, which is potently inhibited by anti-TNF-alpha antibody: a novel mechanism of osteoclastogenesis by IL-17. J Cell Biochem 2009; 108: 947-955.

57 Cochaud S, Giustiniani J, Thomas C, Laprevotte E, Garbar C, Savoye AM *et al*. IL-17A is produced by breast cancer TILs and promotes chemoresistance and proliferation through ERK1/2. Scientific reports 2013; 3: 3456.

58 Hirbe AC, Uluckan O, Morgan EA, Eagleton MC, Prior JL, Piwnica-Worms D *et al*. Granulocyte colony-stimulating factor enhances bone tumor growth in mice in an osteoclast-dependent manner. Blood 2007; 109: 3424-3431.

59 Zhang Q, Chen B, Yan F, Guo J, Zhu X, Ma S *et al*. Interleukin-10 inhibits bone resorption: a potential therapeutic strategy in periodontitis and other bone loss diseases. Biomed Res Int 2014; 2014: 284836.

60 Giordani L, Bruzzi P, Lasalandra C, Quaranta M, Schittulli F, Della Ragione F *et al*. Association of breast cancer and polymorphisms of interleukin-10 and tumor necrosis factor-alpha genes. Clin Chem 2003; 49: 1664-1667.

61 Yang ZP, Ling DY, Xie YH, Wu WX, Li JR, Jiang J *et al*. The Association of Serum IL-33 and sST2 with Breast Cancer. Dis Markers 2015; 2015: 516895.

62 Kim KW, Kim HR, Park JY, Park JS, Oh HJ, Woo YJ *et al*. Interleukin-22 promotes osteoclastogenesis in rheumatoid arthritis through induction of RANKL in human synovial fibroblasts. Arthritis Rheum 2012; 64: 1015-1023.

63 Kim K, Kim G, Kim JY, Yun HJ, Lim SC, Choi HS. Interleukin-22 promotes epithelial cell transformation and breast tumorigenesis via MAP3K8 activation. Carcinogenesis 2014; 35: 1352-1361.

64 Lacey DL, Grosso LE, Moser SA, Erdmann J, Tan HL, Pacifici R *et al*. IL-1-induced murine osteoblast IL-6 production is mediated by the type 1 IL-1 receptor and is increased by 1,25 dihydroxyvitamin D3. The Journal of clinical investigation 1993; 91: 1731-1742.

65 Kim JH, Jin HM, Kim K, Song I, Youn BU, Matsuo K *et al*. The mechanism of osteoclast differentiation induced by IL-1. J Immunol 2009; 183: 1862-1870.

66 Holen I, Lefley DV, Francis SE, Rennicks S, Bradbury S, Coleman RE *et al*. IL-1 drives breast cancer growth and bone metastasis in vivo. Oncotarget 2016; 7: 75571-75584.

67 Gilbert L, He X, Farmer P, Boden S, Kozlowski M, Rubin J *et al*. Inhibition of osteoblast differentiation by tumor necrosis factor-alpha. Endocrinology 2000; 141: 3956-3964.

68 Kobayashi K, Takahashi N, Jimi E, Udagawa N, Takami M, Kotake S *et al*. Tumor necrosis factor alpha stimulates osteoclast differentiation by a mechanism independent of the ODF/RANKL-RANK interaction. The Journal of experimental medicine 2000; 191: 275-286.

69 Madeira MF, Queiroz-Junior CM, Costa GM, Santos PC, Silveira EM, Garlet GP *et al*. MIF induces osteoclast differentiation and contributes to progression of periodontal disease in mice. Microbes Infect 2012; 14: 198-206.

70 Richard V, Kindt N, Saussez S. Macrophage migration inhibitory factor involvement in breast cancer (Review). International journal of oncology 2015; 47: 1627-1633.

71 Schulze J, Bickert T, Beil FT, Zaiss MM, Albers J, Wintges K *et al*. Interleukin-33 is expressed in differentiated osteoblasts and blocks osteoclast formation from bone marrow precursor cells. Journal of bone and mineral research : the official journal of the American Society for Bone and Mineral Research 2011; 26: 704-717.

72 Hu H, Sun J, Wang C, Bu X, Liu X, Mao Y *et al*. IL-33 facilitates endocrine resistance of breast cancer by inducing cancer stem cell properties. Biochem Biophys Res Commun 2017; 485: 643-650.

73 Kamalakar A, Bendre MS, Washam CL, Fowler TW, Carver A, Dilley JD *et al*. Circulating interleukin-8 levels explain breast cancer osteolysis in mice and humans. Bone 2014; 61: 176-185.

74 Mancino AT, Klimberg VS, Yamamoto M, Manolagas SC, Abe E. Breast cancer increases osteoclastogenesis by secreting M-CSF and upregulating RANKL in stromal cells. The Journal of surgical research 2001; 100: 18-24.

75 Ide H, Hatake K, Terado Y, Tsukino H, Okegawa T, Nutahara K *et al*. Serum level of macrophage colony-stimulating factor is increased in prostate cancer patients with bone metastasis. Hum Cell 2008; 21: 1-6.

76 Shukla P, Mansoori MN, Kakaji M, Shukla M, Gupta SK, Singh D. Interleukin 27 (IL-27) Alleviates Bone Loss in Estrogen-deficient Conditions by Induction of Early Growth Response-2 Gene. The Journal of biological chemistry 2017; 292: 4686-4699.

77 Kalliolias GD, Zhao B, Triantafyllopoulou A, Park-Min KH, Ivashkiv LB. Interleukin-27 inhibits human osteoclastogenesis by abrogating RANKL-mediated induction of nuclear factor of activated T cells c1 and suppressing proximal RANK signaling. Arthritis Rheum 2010; 62: 402-413.

78 Lu D, Zhou X, Yao L, Liu C, Jin F, Wu Y. Clinical implications of the interleukin 27 serum level in breast cancer. J Investig Med 2014; 62: 627-631.

79 Kim HJ, Yoon HJ, Yoon KA, Gwon MR, Jin Seong S, Suk K *et al*. Lipocalin-2 inhibits osteoclast formation by suppressing the proliferation and differentiation of osteoclast lineage cells. Exp Cell Res 2015; 334: 301-309.

80 Rucci N, Capulli M, Piperni SG, Cappariello A, Lau P, Frings-Meuthen P *et al*. Lipocalin 2: a new mechanoresponding gene regulating bone homeostasis. Journal of bone and mineral research : the official journal of the American Society for Bone and Mineral Research 2015; 30: 357-368.

81 Oren B, Urosevic J, Mertens C, Mora J, Guiu M, Gomis RR *et al*. Tumour stroma-derived lipocalin-2 promotes breast cancer metastasis. The Journal of pathology 2016; 239: 274-285.

82 Postiglione L, Domenico GD, Montagnani S, Spigna GD, Salzano S, Castaldo C *et al*. Granulocyte-macrophage colony-stimulating factor (GM-CSF) induces the osteoblastic differentiation of the human osteosarcoma cell line SaOS-2. Calcif Tissue Int 2003; 72: 85-97.

83 Park BK, Zhang H, Zeng Q, Dai J, Keller ET, Giordano T *et al*. NF-kappaB in breast cancer cells promotes osteolytic bone metastasis by inducing osteoclastogenesis via GM-CSF. Nat Med 2007; 13: 62-69.

84 Kim KW, Kim BM, Moon HW, Lee SH, Kim HR. Role of C-reactive protein in osteoclastogenesis in rheumatoid arthritis. Arthritis Res Ther 2015; 17: 41.

85 Lehrer S, Diamond EJ, Mamkine B, Droller MJ, Stone NN, Stock RG. C-reactive protein is significantly associated with prostate-specific antigen and metastatic disease in prostate cancer. BJU international 2005; 95: 961-962.

86 Allin KH, Nordestgaard BG, Flyger H, Bojesen SE. Elevated pre-treatment levels of plasma C-reactive protein are associated with poor prognosis after breast cancer: a cohort study. Breast cancer research : BCR 2011; 13: R55.

87 Lean JM, Fuller K, Chambers TJ. FLT3 ligand can substitute for macrophage colony-stimulating factor in support of osteoclast differentiation and function. Blood 2001; 98: 2707-2713.

88 Braun SE, Chen K, Blazar BR, Orchard PJ, Sledge G, Robertson MJ *et al*. Flt3 ligand antitumor activity in a murine breast cancer model: a comparison with granulocyte-macrophage colony-stimulating factor and a potential mechanism of action. Hum Gene Ther 1999; 10: 2141-2151.

89 Panagakos FS. Transforming growth factor--alpha stimulates chemotaxis of osteoblasts and osteoblast-like cells in vitro. Biochem Mol Biol Int 1994; 33: 643-650.

90 Usmani SE, Pest MA, Kim G, Ohora SN, Qin L, Beier F. Transforming growth factor alpha controls the transition from hypertrophic cartilage to bone during endochondral bone growth. Bone 2012; 51: 131-141.

91 Siu MK, Abou-Kheir W, Yin JJ, Chang YS, Barrett B, Suau F *et al*. Loss of EGFR signaling regulated miR-203 promotes prostate cancer bone metastasis and tyrosine kinase inhibitors resistance. Oncotarget 2014; 5: 3770-3784.
